# Department of Therapeutics

**Published:** 1893-07

**Authors:** David Cerna

**Affiliations:** Demonstrator of Physiology in the Medical Department of the University of Texas, etc.; Galveston


### DEPARTMENT OF THERAPEUTICS.

[Under the charge of David Cerna, M. D., Ph. D., Demonstrator of Physiology in the Medical Department of the University of Texas, etc.]

---

HYDROCHLORIDE OF PHENOCOLL.—This new antipyretic has been employed by Kuchalewski (*Kronika Lekarska-Bull. Gener. de Therapeutique*, March 30, 1893) in fifteen patients:—One each of typhoid fever, erysipelas, hepatic colic, chronic myelitis, and facial neuralgia; two of sciatica, and five of articular rheumatism. The author obtained the following results: 1. The drug is a sure antipyretic in doses of from fifty to sixty centigrammes every two hours. 2. In similar cases, phenocoll is an excellent analgesic in certain cases; it also diminishes and often abolishes neuralgic pain. 3. In three out of four cases of chronic articular rheumatism, the use of phenocoll produced a rapid cure. In one case of acute articular rheumatism it did away with the fever, but did not, otherwise, influence the pathological process of the malady. 4. Phenocoll is not suitable for hypodermic use, since it is little soluble in cold water.* 5. The author has

---

*The editor of this department in a recent research, with Dr. W. S. Carter (Notes on New Remedies, New York, September, 1892,) found the hydrochloride of phenocoll quite soluble in water.

not seen untoward effects produced by phenocoll even when administered in as high doses as three grammes in the twenty-four hours; neither has it caused disturbances of digestion or of any of the other organs. The urine has often appeared of an intense black color, but it has failed, nevertheless, to respond to Hertl's reaction.

---

CAFFEINE IN ALCOHOLISM.—Czarkowski (*Gazette Lakarska-Bull. Gener. de Therapeutique*, April 30, 1893) calls attention to the untoward effects of caffeine in the treatment of alcoholic patients. Based upon four personal observations, the author regards the use of caffeine as contra-indicated in alcoholics. In these instances the drug produced cerebral excitement, this disappearing only on discontinuing the administration of the alkaloid. The first case was that of a patient suffering from mitral insufficiency with œdema, in which the excitement was marked after the ingestion of two grammes of the citrate of caffeine, given in the course of twenty-four hours. On stopping the medicament the patient became quiet, but did not remember anything that had occurred during the period of excitation. In the second case, that of a nephritic patient, the cerebral disturbance came on after the fifth dose of twenty centigrammes of the same salt. After the intense excitement there followed a period of quietude accompanied with a loss of consciousness lasting several hours. The third case was one of typhus. In this the influence exercised by several doses, of sixty centigrammes each, was that of a furious delirium, accompanied with a homicidal tendency, a disturbance of which the patient had no knowledge afterwards. The author concludes that in alcoholic patients, the use of caffeine requires discrimination, and that the drug should be employed at the beginning only in small doses, discontinuing the ingestion of the medicament on the appearance of the slightest excitement.

---

TIN-PLATE TREATMENT OF ULCERS,—Robert Coltman (*The Universal Medical Journal*, April, 1893) has treated four cases of chronic ulceration on the side of the leg by the above method. Cases had all resisted other plans of treatment for several months. The ulcers were washed with 1 to 1000 solution of bi-chloride; he dried the cavity with antiseptic cotton and then filled it with boracic acid, and covered it with a piece of patent lint one-half inch wider than the margins of the ulcer; then fitted a piece of ordinary tin can upon the lint and bound the limb tightly with roller bandages. Improvement was immediate; dressings were removed each third day, and, after washing out the ulcer with hot boracic acid solution, they were re-applied. About three weeks sufficed for a cure.

---

BELLADONNA IN INCARCERATED HERNIA.—V. B. Zagorsky (*Meditzin-Obozren—University Medical Magazine*, May, 1893) reports five cases incarcerated hernia—four inguinal and one umbilical—in which, after taxis had failed, extract of belladonna, in doses of one-fourth of a grain every hour, was followed by spontaneous reduction. The result was attributed to the antispasmodic property of the drug.

---

TREATMENT OF PSORIASIS.—In an excellent report of the treatment of psoriasis, Henry W. Stelwagon (*University Medical Magazine*, May, 1893) makes the following authoritative statement: For the general treatment the chief remedies were arsenic, liquor potassæ, potassium iodide, potassium acetate, tar and oil of copaiba; and in certain cases tonics, especially cod-liver oil. The most valuable of these were arsenic aud liquor potassæ, the latter being particularly so in the markedly inflammatory type occurring in robust patients. Arsenic was an exceedingly useful remedy in first attacks and in patients in whom the disease had been of long duration but who had never been systematically treated; in recurrences and in patients who had previously taken the drug freely, it was without much, if any, value. In the external treatment, plain or alkaline baths, chrysarobin, pyrogallic acid, tar, beta-naphthol, aristol and white precipitate were variously employed. In certain cases of mild type of eruption, even though more or less extensive, a daily alkaline bath, followed by a tolerably vigorous application of a salicylated ointment, (10 to 30 grains to the ounce), often sufficed to remove the disease. Chrysarobin was used either as an ointment or collodion paint, 5 to 20 per cent. strength; pyrogallic acid, as an ointment or paint, 5 to 10 per cent. strength; tar, either as the officinal tar ointment, full strength or weakened, or as an oil, for this latter purpose using the oil of cade, either pure or with one or two parts of alcohol; beta-naphthol, as an ointment, 5 to 15 per cent. strength; and white precipitate, as an ointment, 40 to 100 grains to the ounce. Tar was the most generally applicable and useful external remedy; chrysarobin was valuable and often rapidly curative, but it has certain known disadvantages, which make it an unsafe remedy for general use in this class of patients. In conclusion, according to the extensive experience of the author, it may be said that those giving the best results were tar, chrysarobin, and white precipitate; beta-naphthol and aristol, while useful in some cases, did not compare in value to the others named.

---

SYPHILITIC FEVER.--Two cases of syphilitic fever are described by J. P. Gayon (*La Escuela de Medicina*, Mexico, Jan. 31, 1893). The first patient was a young man who became affected with a well marked phagadenic white chancre of the penis, quite painful to the touch. The resulting adenitis had arrived at the stage of suppuration when the case was first seen. Proper cauterization with nitric acid, thorough antisepsis, and the local application of iodoform, produced in the course of time a complete cure. Twenty days afterwards, however, the patient was attacked by an intense chill followed by a rise of temperature to 40° C. (104°F.), accompanied with cephalalgia, coated tongue, general malaise, and various other symptoms peculiar to a febrile disorder. A thorough examination failed to reveal affection of any of the organs. The fever, with slight morning remissions, remained at the height referred to during three days, and then a pustular eruption, similar to that of small-pox, made its appearance upon the skin, and, particularly upon the mucous membrane of the throat. The history of the case made the diagnosis easy. The patient was evidently suffering now from secondary syphilis. Under specific treatment the fever and all other symptoms quickly disappeared. In the other case the fever assumed an intermittent type, and believing it at first to be one of malarial origin, quinine was administered freely, but this medicament failed to subdue the abnormal bodily temperature. Under specific treatment a cure was again produced as in the former case. According to the author, therefore, syphilis may manifest itself alone by an access of fever, which, as in tuberculosis for example, may assume an intermittant type (hectic) or a continued typhoid form. In both instances, one or the other type of the febrile phenomenon may be due directly to the amount of the toxine absorbed.

